# Dielectrophoretic Characterization of Tenogenically Differentiating Mesenchymal Stem Cells

**DOI:** 10.3390/bios11020050

**Published:** 2021-02-16

**Authors:** Anthony T. Giduthuri, Sophia K. Theodossiou, Nathan R. Schiele, Soumya K. Srivastava

**Affiliations:** Department of Chemical & Biological Engineering, University of Idaho, Moscow, ID 83844-1021, USA; gidu3424@vandals.uidaho.edu (A.T.G.); theo4146@vandals.uidaho.edu (S.K.T.); nrschiele@uidaho.edu (N.R.S.)

**Keywords:** dielectrophoresis, dielectric properties, mesenchymal stem cells, tendons, tenogenesis

## Abstract

Tendons are collagenous musculoskeletal tissues that connect muscles to bones and transfer the forces necessary for movement. Tendons are susceptible to injury and heal poorly, with long-term loss of function. Mesenchymal stem cell (MSC)-based therapies are a promising approach for treating tendon injuries but are challenged by the difficulties of controlling stem cell fate and of generating homogenous populations of stem cells optimized for tenogenesis (differentiation toward tendon). To address this issue, we aim to explore methods that can be used to identify and ultimately separate tenogenically differentiated MSCs from non-tenogenically differentiated MSCs. In this study, baseline and tenogenically differentiating murine MSCs were characterized for dielectric properties (conductivity and permittivity) of their outer membrane and cytoplasm using a dielectrophoretic (DEP) crossover technique. Experimental results showed that unique dielectric properties distinguished tenogenically differentiating MSCs from controls after three days of tenogenic induction. A single shell model was used to quantify the dielectric properties and determine membrane and cytoplasm conductivity and permittivity. Together, cell responses at the crossover frequency, cell morphology, and shell models showed that changes potentially indicative of early tenogenesis could be detected in the dielectric properties of MSCs as early as three days into differentiation. Differences in dielectric properties with tenogenesis indicate that the DEP-based label-free separation of tenogenically differentiating cells is possible and avoids the complications of current label-dependent flow cytometry-based separation techniques. Overall, this work illustrates the potential of DEP to generate homogeneous populations of differentiated stem cells for applications in tissue engineering and regenerative medicine.

## 1. Introduction

Mesenchymal stem cells (MSCs) are multipotent cells that can self-renew and differentiate into various cell lineages such as adipocytes [1], chondrocytes [2], osteoblasts [3,4], myocytes [1,5], and tendon cells [6,7]. Since their discovery in the 1960s [8], MSCs have been gradually established as a standard cell source in the field of regenerative medicine [9]. MSCs can be isolated from bone marrow [10], adipose tissue, skin, peripheral blood, and perinatal tissues such as umbilical cord blood, amniotic fluid, fetal membrane [11,12], and placenta [13]. MSCs are promising for regenerative therapies due to their relative ease of isolation and multipotency [14]. MSCs have been explored to treat several conditions related to cardiovascular health [15,16,17] and other chronic conditions such as lupus, diabetes mellitus, liver cirrhosis, and Crohn’s disease [18]. As of 2016, at least 493 MSC-based clinical trials have been completed or are ongoing [19].

MSCs have also been explored in tissue engineering and regenerative approaches for treating musculoskeletal tissue injuries, including tendon injuries [7,20,21,22,23,24,25]. Tendons are musculoskeletal tissues composed primarily of collagen type I that transfer forces from muscle to bone to enable movement. Tendons are frequently injured, and the clinical options for treating tendon injuries are limited, motivating the development of MSC-based therapies. As with most stem cells, the inherent heterogeneity of MSC populations limits their current clinical use. MSCs are heterogeneous in terms of differentiation potential and size, with cell diameters ranging widely from 15 to 50 μm [18,26]. This considerable variance in size is known to be the cause for severe vascular obstructions and stroke in animal models [27], thus limiting the clinical uses of MSCs in human patients. Undifferentiated stem cells that remain within the differentiating population can result in abnormal tissue formation and differentiation, such as ectopic ossification when used in tendon repairs [28,29], or form malignant tumors [30]. Overall, improved stem cell characterization and separation techniques to achieve precise control over stem cell differentiation are needed before MSC-based regenerative therapies can be reliably used clinically.

Current stem cell separation techniques can be classified into two categories: techniques based on physical parameters such as size and density (i.e., density gradient centrifugation, field-flow fractionation, etc.) and techniques based on affinity (i.e., on chemical, electrical, or magnetic couplings) such as fluorescence-activated cell sorting (FACS) [31] and magnetic-activated cell sorting (MACS) [32]. While effective, size/density-based separation techniques are time-consuming, expensive, require prior knowledge of the target cell type’s size/density parameter [33], and cannot separate cells of the same density and size. Affinity-based methods (FACS and MACS) were developed to overcome these limitations. Although effective, these techniques involve labeling the cells with antibodies tagged with fluorescent dyes or magnetic beads (for MACS). Additionally, both FACS and MACS require tedious cell preparation and instrumentation protocols [34], are labor-intensive, and have high operating costs [33,35]. Finally, these labeling techniques may alter cellular function, which is problematic for cells used in regenerative therapies [18]. Overall, there is a significant need for accurate, cost-effective, and efficient stem cell separation techniques to generate homogeneous populations of stem cells for tissue engineering and regenerative medicine applications.

This article demonstrates the use of dielectrophoresis (DEP), an electrokinetic technique, to identify the unique dielectric properties of tenogenically differentiating MSCs from larger cell populations in a label-free way. DEP employs non-uniform electric fields and exploits the effects of the electric fields on cellular motion to characterize and separate them. DEP-based sorters are simple, cost-effective, label-free, accurate, and efficient, overcoming the limitations of current commercial stem cell separation methods. DEP was first used in stem cell research in the 1990s [36,37,38] and has progressed significantly over the past two decades in terms of separation accuracy and efficiency, leading to renewed interest in DEP as a tool to characterize stem cells and their differentiated progeny [14,39].

The work presented here is the primary step toward developing DEP as a novel, label-free sorting technique for baseline (i.e., undifferentiated) and tenogenically differentiating MSCs, based on the dielectric characterization of both the cell membrane and cytoplasm at different stages of early differentiation. Stem cell therapy is seen as a potential method to heal tendon injuries and is capable of being clinically translated. However, due to the limited understanding of tenogenesis and post-culture processing limitations, clinical translation had been delayed for many years [40]. Hence, there is a research need to characterize and identify tenogenic differentiation potential and also a technique that distinguishes undifferentiated MSCs from their differentiating progeny. Here, we evaluate DEP, a powerful technique for cell characterization and separation, which had been in practice for several decades [14]. However, to our knowledge, no studies have used DEP to characterize the tenogenesis of MSCs. We show that DEP can identify undifferentiated MSCs from their differentiating progeny based on small changes in the electrical properties of the cells. This study highlights DEP’s potential use for identifying tenogenically differentiating MSCs in a label-free way, which can ultimately be used to generate homogeneous stem cell populations for enhanced tendon regeneration.

### 1.1. Theory of DEP

DEP is the force observed acting on dielectric particles subjected to a non-uniform alternating current (AC) electric field due to the difference in the polarizability of the medium and the particles [41,42]. For a spherical particle of radius r, the magnitude of DEP force is given as

(1) F→DEP=2πr3εoεmRe[K(ω)]∇E2

where $\varepsilon_{o}$ and $\varepsilon_{m}$ are the permittivity of the free space and the relative permittivity of the surrounding medium, respectively, Re[K(ω)] is the real part of the Clausius–Mossotti factor, which is discussed below in Equation (2), and $\nabla E^{2}$ signifies the gradient of an electric field. The force acting on the cells can be tuned by adjusting the AC frequency and magnitude. Cell motion due to the force acting on the cells under the electric field is defined by the Clausius–Mossotti factor, K(ω), which is given by:

(2) $$K\left(\omega \right)=\frac{\varepsilon_{\mathrm{cell}}^{*}-\varepsilon_{\mathrm{med}}^{*}}{\varepsilon_{\mathrm{cell}}^{*}+2\varepsilon_{\mathrm{med}}^{*}}$$

where $\varepsilon_{\mathrm{cell}}^{*}$ and $\varepsilon_{\mathrm{med}}^{*}$ are the complex permittivities of the cell and the medium, respectively. Complex permittivity, $\varepsilon^{*}$ is defined as:

(3) $$\varepsilon^{*}=\;\varepsilon -j\frac{\sigma }{\omega }$$

where ε is permittivity, σ is conductivity, and ω is the angular frequency of the applied electric field. For spherical particles, Re[K(ω)] ranges between −0.5 and 1, accounting for the particle’s polarizability [41,43]. Further estimation of dielectric properties using the determined complex permittivities of the membrane and the cytoplasm, to calculate $\varepsilon_{\mathrm{cell}}^{*}$, and thereby K(ω) using Equation (2) requires an appropriate shell model. Biological shell models can be classified into single, double, multi-shell models, and ellipsoidal models discussed elsewhere [44,45]. In this study, MSCs are modeled using a single shell model.

### 1.2. Single Shell Model

**Figure 1:**
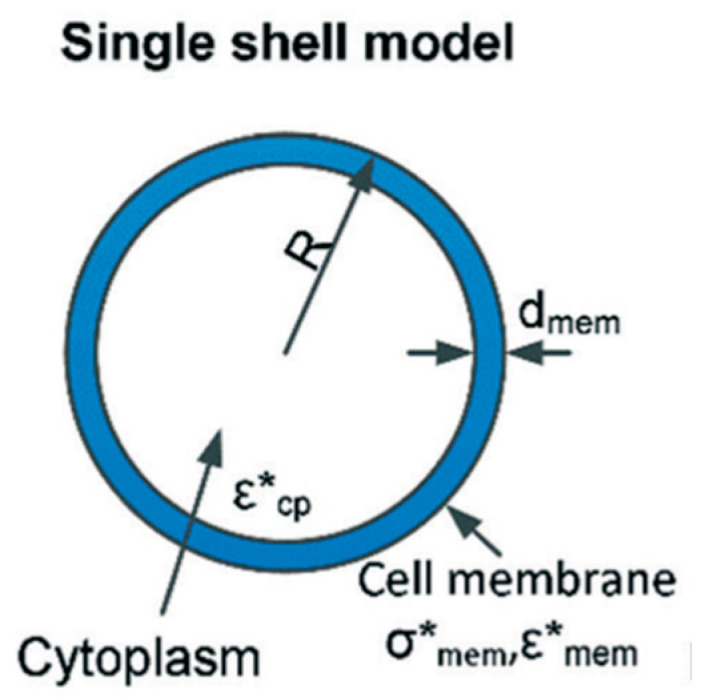
Single shell model of a cell where ε and σ denote permittivity and conductivity, respectively. Subscripts mem and cp refer to the properties of the cell membrane and cytoplasm, respectively [44].

Stem cells’ cytoplasm and its contents, i.e., nucleus, DNA, organelles, etc., are considered one homogenous sphere surrounded by a plasma membrane to reduce complexity (Figure 1). For a single shell model, the complex permittivity of a cell is given by [45,46]:

(4) $$\varepsilon_{\mathrm{cell}}^{*}=\varepsilon_{\mathrm{mem}}^{*}\left[\frac{{\left(\frac{R}{R-d}\right)}^{3}+2\left(\frac{\varepsilon_{\mathrm{cp}}^{*}-\varepsilon_{\mathrm{mem}}^{*}}{{\;\varepsilon }_{\mathrm{cp}}^{*}+2\varepsilon_{\mathrm{mem}}^{*}}\right)}{{\left(\frac{R}{R-d}\right)}^{3}-\left(\frac{\varepsilon_{\mathrm{cp}}^{*}-\varepsilon_{\mathrm{mem}}^{*}}{\varepsilon_{\mathrm{cp}}^{*}+2\varepsilon_{\mathrm{mem}}^{*}}\right)}\right]$$

where R is the outer radius of the cell, *d* is the thickness of the membrane, and the subscripts mem and cp refer to the cell membrane and cytoplasm, respectively.

DEP force is dependent on the frequency of the applied electric field. As frequency is tuned by maintaining a fixed peak-to-peak voltage, cells exhibit distinct behavior based on their polarizability. When the cell’s polarizability is greater than the polarizability of the medium in which it is suspended, the cell is attracted toward the high field electrode; this is termed as positive dielectrophoresis (pDEP). When the cell’s polarizability is less than that of the medium, the cell moves away from the high field electrode, thus experiencing a force termed as negative dielectrophoresis (nDEP) [47]. There exists a particular frequency at which the cell experiences no net DEP force, which is termed the crossover frequency [48]. Cells typically display two crossover frequencies. The lower crossover frequency ($f_{x1}$), also referred to as the first crossover frequency, occurs in the β region (kHz to several MHz), where cells transition from nDEP to pDEP. This first crossover frequency $\left(f_{x1}\right)$ depends on cell size, shape, and the outer membrane physiology [18]. The higher crossover frequency $(f_{x2}),$ also referred to as the second crossover frequency, occurs at frequencies above 10 MHz in a low-conductivity medium [18], where cells transition from pDEP back to nDEP. The second crossover frequency depends on changes in the cell’s interior physiology, particularly those associated with the nucleus and the nuclear size compared to overall cell volume. Additionally, changes in the conductivity of the suspending medium do not impact the $f_{x2}$ [49] but impact $f_{x1}$, where $f_{x1}$ is directly proportional to the medium conductivity [48].

A direct relation correlating $f_{x1}$ with the membrane capacitance, $C_{\mathrm{mem}}$, and medium conductivity is given by [18,43]:

(5) $$f_{x1}=\frac{\sqrt{2}\sigma_{m}}{2{\pi \mathrm{rC}}_{\mathrm{mem}}}.$$

Using $C_{mem}$, calculated from Equation (5), the permittivity of the membrane ($\varepsilon_{mem}$) can be obtained using the relation:

(6) $$\varepsilon_{mem}=\frac{C_{mem}d}{4\pi r^{2}\varepsilon_{0}}$$

where ‘*d*’ is the thickness of the membrane, and $\varepsilon_{0}$ is the permittivity of the vacuum/free space—8.854 × 10^−12^ F/m. To estimate the dielectric properties of the cell interior (cytoplasm), the following relation is used as given by Gimsa et al. [48]:

(7) $$f_{x2}^{2}=\frac{1}{4\pi^{2}}\frac{1}{\varepsilon_{o}^{2}}\frac{\left(\sigma_{mem}-\sigma_{cp}\right)\left(\sigma_{cp}+2\sigma_{mem}\right)}{\left(\varepsilon_{cp}-\varepsilon_{mem}\right)\left(\varepsilon_{cp}+2\varepsilon_{mem}\right)}$$

Using the equations listed above, the dielectric properties of the membrane and cytoplasm can be estimated using the experimentally determined crossover frequencies. However, further optimization of these estimated dielectric parameters is required and is achieved through curve fitting and non-linear regression, where initial estimates of dielectric properties are used to back-calculate the crossover frequency (labeled theoretical) and then optimized by minimizing the sum of squares error using non-linear regression. The properties estimated can then be used to generate the DEP characteristic curve for determining the optimum sorting region or the region of frequency where cells can be separated effectively without damaging the cells (i.e., without high-frequency exposure).

## 2. Materials and Methods

### 2.1. Overview

In this study, murine MSCs were cultured for baseline, 0 (switched to a low-serum medium), 3, and 7 days (d). The dielectric properties of undifferentiated MSCs (i.e., no treatment controls) were compared to MSCs treated with the transforming growth factor beta (TGFβ)2 (i.e., treatment). TGFβ2 has previously been shown to induce tenogenesis in MSCs [50,51]. Following the culturing process, cells were suspended in DEP medium, and crossover frequency experiments were conducted to quantify the dielectric parameters of baseline, tenogenically differentiating, and control MSCs. Experimental results were statistically analyzed using Prism 8, and cell diameter was also quantified using Image J. Finally, curve fitting and best-fit estimates were generated to determine the permittivity and conductivity of the cells.

### 2.2. Cell Culture

Murine MSCs (C3H10T1/2, ATCC, Manassas, VA, USA), an immortalized cell line used in previous studies exploring tenogenesis [52,53], were cultured and augmented with the tenogenic growth factor TGFβ2, as described elsewhere [50]. Briefly, MSCs were expanded in standard growth medium (Dulbecco’s Modified Eagle’s Medium (DMEM), 10% fetal bovine serum (FBS), and 1% penicillin/streptomycin) until 70% confluent. Cells were used between passage 3 and 9. Using trypsin, MSCs were detached and seeded into each well of a 6-well plate at 5000 cells/cm^2^. To allow for initial cell attachment, MSCs were incubated for ≈24 h. Then, MSCs were washed with warm phosphate-buffered saline (PBS) (Gibco, Grand Island, NY, USA), the medium was changed to low-serum DMEM (DMEM, 1% FBS, 1% penicillin/streptomycin), and the cells were incubated for ≈24 h to allow equilibration. Following equilibration, the cells were rinsed again with warm PBS and cultured for 0, 3, or 7 days (d) in the low-serum medium with a corresponding amount of sterile water (vehicle controls) or with 50 ng/mL recombinant human TGFβ2 (3 and 7 d timepoints only) (PeproTech, Rocky Hill, NJ, USA). The medium was changed every third day. To evaluate cell morphology with TGFβ2-induced tenogenesis, cells were also cultured as described above but on glass coverslips. Cells were fixed overnight in 10% formalin at 4 °C, permeabilized with 0.1% Triton X-100 (Acros Organics, Fair Lawn, NJ, USA), and stained with Fluorescein isothiocyanate (FITC)-phalloidin and 4,6-diamidino-2-phenylindole (DAPI) (Life Tech., Waltham, MA, USA) to observe the actin cytoskeleton and cell nuclei, respectively. Mounted coverslips were imaged on a spinning-disk confocal microscope (Nikon/Andor, Melville, NY, USA).

To collect the cells for DEP characterization experiments, cells were washed in warm PBS and trypsinized for 3 min to ensure cell detachment from the well. Cells collected at day 7 required slightly prolonged trypsinization (up to 5 min) to detach them from the well. The trypsin was neutralized using a low-serum medium, and cells were centrifuged at 1200 revolutions per minute (RPM) for 8 min. The supernatant was discarded, and the cell pellet was resuspended to approximately 10^6^ cells/mL in the DEP solution medium (described in Section 3.3) of a known standard, which served as the medium for the DEP experiments. The suspended cell pellet obtained was used for DEP experiments within 30 min of trypsinization. Experiments were repeated a minimum of 3 times for each time point.

### 2.3. DEP Suspending Medium

The conductivity of the DEP suspending medium is one of the critical parameters that affect the experimental cellular response to the first crossover frequency. Ideally, the medium is maintained at isotonic conditions to avoid shrinking, swelling, or lysis of the suspended cells. A standardized DEP suspending medium (50 g/L) was prepared by dissolving 1.25 g of D-glucose in 25 mL of deionized (DI) water. The pH of the suspending medium was maintained between 6.5 and 7. The conductivity of the medium was adjusted to ≈0.060 S/m using sodium chloride crystals. The conductivity of the DEP solution was kept constant, and all the experiments were performed at room temperature (21 °C).

### 2.4. DEP Experimental Setup

**Figure 2:**
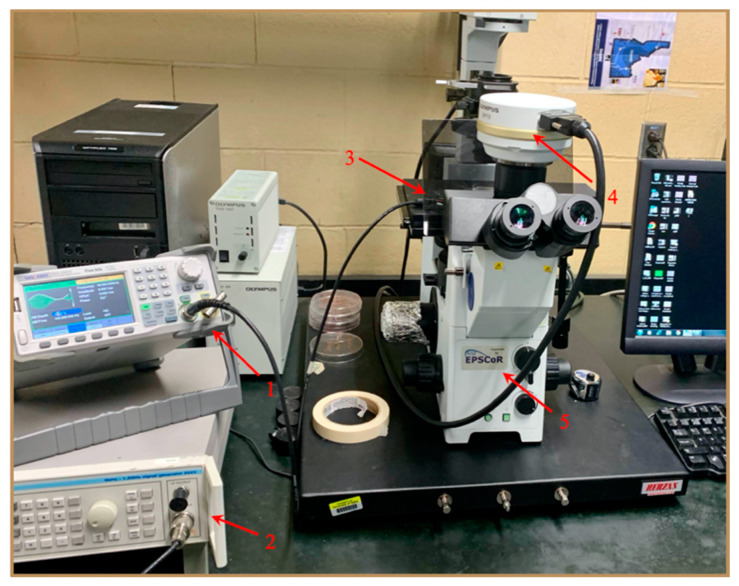
Image showing the experimental setup where the labeled parts refer to (**1**) function generator (up to 80 MHz) for $f_{x1}$ experiments, (**2**) function generator (up to 1200 MHz) for $f_{x2}$ experiments, (**3**) stage on which the dielectrophoretic (DEP) microwell is placed, (**4**) camera to record/visualize the experiments, and (**5**) Olympus IX-71 Inverted Microscope.

The physical DEP experimental setup requires a microscope to monitor the cellular movement changes as the frequency is tuned, a DEP microwell to suspend the cells, and a function generator to tune the AC frequency. The experimental setup for this study is shown in Figure 2. The primary step in DEP experiments is the fabrication of the microwell using the soft-lithography technique. This technique is termed “soft” due to the usage of elastomeric polymers in fabricating the microwell. The post-fabrication steps involve the electrode setup and sealing the device with appropriate spacing. This process is discussed in detail below.

#### 2.4.1. Microwell Fabrication

**Figure 3:**
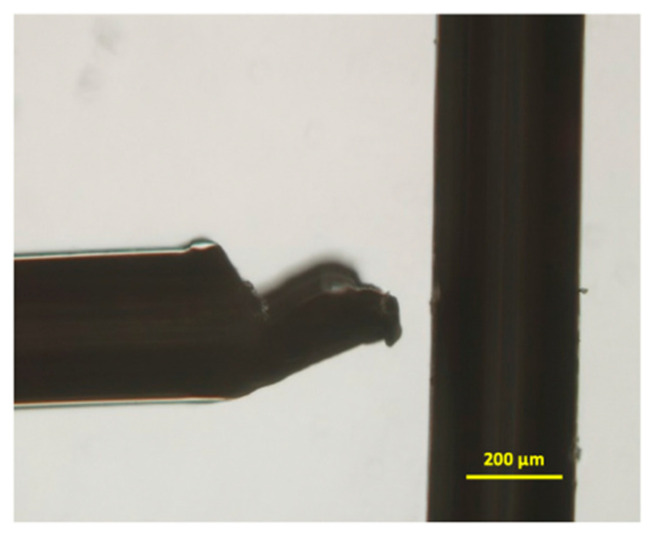
Sample image showing sealed platinum electrode setup for point and planar microwell with ≈75 µm electrode spacing between them.

A point-and-planar electrode DEP microwell device was fabricated as previously described [54,55], with slight modifications to handle stem cells. Briefly, ≈30 g of Sylgard 184 silicone elastomer (Dow Corning, Midland, MI, USA) was weighed in a 50 mL glass beaker using an analytical balance (XS204, Mettler Toledo, Columbus, OH, USA) and mixed with the accompanying curing agent (≈3 g) at a 10:1 ratio. Then, the mixture was rapidly mixed with a glass rod and then degassed in a vacuum chamber to ensure that all the air incorporated in the mix due to stirring was removed, yielding an air-free, bubble-free mixture. The clear mixture was then poured into a polystyrene petri dish (100 mm × 15 mm) placed inside a dust-free, sanitized biosafety cabinet so that no dust gets into the polymer mix. Then, the polymer solution was cured at 75 °C. The cured poly(dimethylsiloxane) (PDMS) was cooled and cut into ≈6 mm × 6 mm pieces with precision, followed by a microwell, punched with a 3 mm Miltex biopsy punch, and then cut into square-shaped PDMS pieces. The PDMS piece with a punched microwell was plasma cleaned using our in-house microwave plasma cleaner [56] for 2 min and sealed onto a pre-cleaned micro slide (25 mm × 75 mm, 1 mm thick) by inverting the plasma exposed PDMS surface onto the micro glass slide in less than 10 s after removal from the plasma cleaner. Then, the device is left undisturbed for ≈30 min to seal firmly and form a leak-free microwell platform. A high-grade platinum (Pt) wire (99.5%, 0.2 mm diameter) was cut into 15-mm pieces and inserted perpendicularly into the microwell. The Pt electrodes were approximately on the same plane as that of the micro slide. These Pt wires serve as the electrodes and deliver a non-uniform electric field gradient for the DEP crossover frequency experiments. A simplified figure showing the electrode setup is included in Figure 3. Spacing between the electrodes was adjusted to ≈75 µm by using an inverted microscope (Olympus IX-71). The electrode wires were sealed using epoxy to permanently fix the distance between the two electrodes, i.e., point and planar electrodes. Occasionally, the spacing was altered due to the pipette’s in and out motion from the microwell (i.e., when rinsing the microwell); when this occurred, the spacing was adjusted back to the initial spacing. The electrode distance was verified and re-measured at the beginning of every experiment.

#### 2.4.2. DEP Crossover Frequency Experiments

The platinum electrodes in the microwell were connected to a waveform function generator (Siglent SDG 2082X), and sinusoidal signals at a voltage of 8 V_pp_ were supplied to the microwell to create the non-uniform electric field. Frequency was swept until the lower crossover frequency, $f_{x1}$, was found, where no apparent motion was seen in the cells. The specific function generator used, the SDG 2082X, cannot sweep frequencies higher than 80 MHz, and thus, the higher crossover frequency, $f_{x2}$, experiments were completed using a different function generator (Marconi Instruments, 9 kHz to 1.2 GHz signal generator 2023) at 13-decibel milliwatts (dBm) equivalent to ≈2.825 V_pp_ to experimentally determine the higher crossover frequency where the second transition of pDEP back to nDEP occurred. Once the frequency range for transitions were determined, the frequency was further fine-tuned to obtain the single frequency (integer) value. Experiments were repeated for three different cell samples at every timepoint (biological replicates) at least nine times (technical replicates) for all the stem cell groups (e.g., undifferentiated baseline MSCs, tenogenically differentiating MSCs (treatment group), undifferentiated MSCs (no treatment-control group)) to determine the average crossover frequencies (*f_x_*_1_ and *f_x_*_2_) at a single DEP suspending medium conductivity (≈0.06 S/m).

DEP crossover frequency response of the undifferentiated (baseline control), undifferentiated at days 3 and 7 (no treatment control), and tenogenically differentiating MSCs at days 3 and 7 (TGFβ2-treatment) were recorded after suspending the cells in the DEP medium of conductivity 0.06 S/m. At a fixed AC voltage of 8 V_pp_, the frequency was swept from 0.01 to 0.5 MHz in increments of 0.005 MHz and from 1 to 300 MHz in increments of 5 MHz to record $f_{x1}$ and $f_{x2}$, respectively. All experiments were completed within 30 min of suspending the cells in the DEP suspending medium, and cells were exposed to the AC electric field for no longer than 1 min at each frequency point. Following 1 min of cell exposure, the microwell was rinsed thoroughly with DEP suspending medium, and 2 μL of fresh cell suspension from the same sample aliquot was pipetted into the microwell to perform the technical replicate experiments (9 times). This protocol was followed for all the cell groups. In general, mammalian cells exhibit nDEP at low frequencies and pDEP at higher frequencies, within the frequency range of 0.01–1 MHz [57], i.e., the β-region. A similar trend was observed while characterizing dielectric differences in murine cells under normal and cancerous conditions [58]. To avoid spatial movement, which is 3D dielectrophoresis, the current study involves only measuring the crossover frequency in two-dimensional ways by limiting the non-uniformity of the XY plane by aligning the electrodes as close to the glass slide surface as possible. Every trial had ≈2000 cells added to the microwell (1 × 10^6^ cells/mL).

### 2.5. Statistical Analysis

$f_{x1}$ and $f_{x2}$ obtained through these DEP crossover frequency experiments were analyzed separately using one-way analysis of variance (ANOVA) and Welch’s t-test (Prism 8, GraphPad, San Diego, CA, USA). All reported values are expressed as mean ± standard error of the mean (SE). All the values reported are based on multiple runs for every group.

### 2.6. Image Processing

Images captured during experiments were analyzed using ImageJ [59] to measure cell size within the different treatment groups. Cell diameter was measured manually by setting up the scale and using a straight segmented line. The area of the cells was also measured using the embedded ‘Analyze Particle’ tool. At least six images corresponding to the crossover experiments were analyzed for each treatment group, and their measurements were averaged to obtain the mean cell radius.

### 2.7. Curve-Fitting Procedure

After obtaining experimental crossover frequencies and initial estimates for permittivity and conductivity, the crossover frequency was theoretically estimated using Equations (5) and (7) and adjusted using non-linear regression to minimize the residual sum of squares error (difference between experimental and theoretical crossover frequencies) using Microsoft Excel. Initial estimates for the parameters were calculated using Equations (5) and (6) for the membrane characteristics, while initial estimates of the cytoplasmic properties were obtained from the literature [18]. Once an appropriate estimate was obtained, the sum of squares was minimized using an optimization algorithm by changing the estimated values until the error was minimized. The values were considered final and best-fit estimates at the least possible sum of squares error; these are considered the optimized values.

## 3. Results and Discussion

### 3.1. DEP Crossover Frequency Response of MSCs

**Figure 4:**
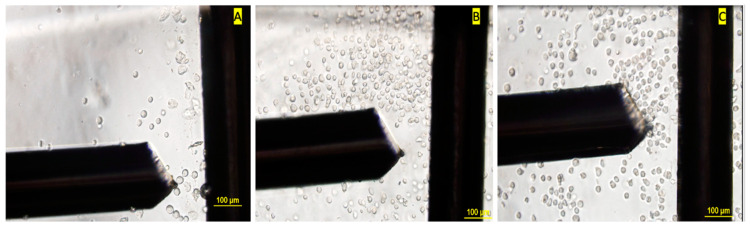
Images describing movement of mesenchymal stem cells (MSCs) away and toward the high electric field region, i.e., negative dielectrophoresis (nDEP) and positive dielectrophoresis (pDEP) for different cell groups. (**A**) Baseline cells–control group experiencing pDEP at 105 kHz and 8 V_pp_, (**B**) 3 day–no treatment group experiencing nDEP at 110 kHz and 8 V_pp_, and (**C**) 3 d treatment group with transforming growth factor beta 2 (TGFβ2), i.e., differentiating into tendon cells experiencing pDEP at 200 kHz and 8 V_pp_.

Mean first crossover frequencies were observed to be 0.0986 ± 0.0003 MHz, 0.134 ± 0.003 MHz, and 0.175 ± 0.007 MHz for 0 d control, 3 d–no treatment control, and 3 d–TGFβ2 treatment groups, respectively, at 0.06 S/m medium conductivity and an AC voltage of 8 V_pp_ (Table 1). Analysis of categorical independent variables (three different cell groups at 3 d) and numerical dependent variable ($f_{x1}$) using one-way ANOVA showed significantly different crossover frequencies between groups (*p* < 0.05). Unpaired *t*-tests with Welch’s correction showed significant differences in $f_{x2}$ between undifferentiated MSCs and tenogenically differentiating MSCs at 3 d (*p* < 0.05). However, 0 d baseline control MSCs and the undifferentiated 3 d controls were not statistically significant, meaning that they had similar $f_{x2}$, indicating possible similarities in the electrophysiology of their interior/cytoplasm. This indicates that the cell’s interior dielectric properties of 0 d baseline control MSCs do not differ significantly from those of undifferentiated control MSCs cultured for 3 days with vehicle controls and without TGFβ2 treatment. The movement of cells from the electrode when exposed to different frequencies at 8 V_pp_ was observed. Unique nDEP and pDEP behaviors were observed between groups (Figure 4), specifically baseline control MSCs exhibited pDEP at 105 kHz, 3 d undifferentiated controls exhibited nDEP at 110 kHz, and tenogenically differentiating MSCs at 3 d exhibited pDEP at 200 kHz.

Interestingly, at 3 days, significant differences were detected in the first crossover frequencies of the no treatment controls and tenogenically differentiating MSCs. This finding suggests that an early application of DEP can detect small differences in dielectric properties between tenogenically and non-tenogenically differentiating MSCs.

Untreated control MSCs at 0 and 7 d had first crossover frequencies ranging from 200 to 210 kHz and 135 to 150 kHz, respectively, at 0.06 S/m and 8 V_pp_. However, for 0 and 7 d treated MSCs, no significant differences were detected in crossover frequency, as the experimental values were obtained for $f_{x1}$ and $f_{x2}$ were not consistent between runs (Table 1).

**Figure 5:**
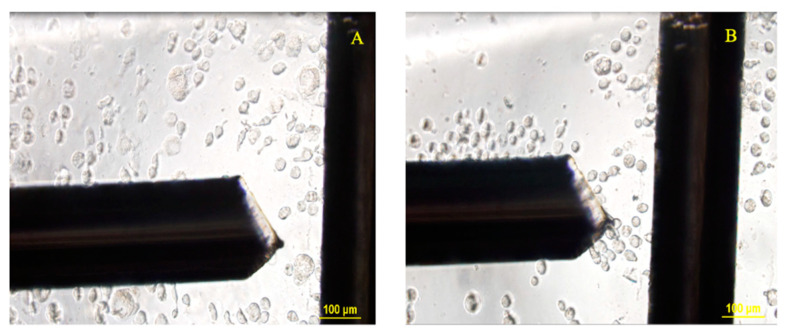
Images showing movement of cells away from and toward the high electric field region, i.e., nDEP and pDEP for 7-day cell groups. (**A**) Treated cells appear to be elongated compared to (**B**) untreated cells. Notably, the degree of elongation varied even though the experiments were at the same timepoint.

Several factors may account for the variability in experimental values. On day 0, MSCs had not been treated with TGFβ2 but instead were immediately collected for sorting to determine any impacts of the 24 h equilibration in low-serum medium on the cellular dielectric properties. Thus, cells at day 0 represent a highly heterogeneous MSC population with varied characteristics, such that no single first and second crossover frequency emerge. Additionally, as expected, baseline and day 0 cells did not significantly differ from one another in terms of crossover frequencies (*p* > 0.1). Conversely, by day 7, cells appeared more elongated (Figure 5) with crossover values of 110–130 kHz for smaller elongated cells, while the large ones had values ranging between 50 and 70 kHz.

Crossover frequency for the treated MSCs groups at day 7 did not differ significantly from the 0 d groups. This may be due to some cells having differentiated considerably by day 7, as evidenced by changes in morphology and the need for longer trypsinization to disrupt the cell adhesions prior to DEP separation. Various differentiation stages within a single MSC population are expected, but the heterogeneity may mask the signature dielectric characteristics of the tenogenically differentiating cells at 7 d. Overall, the 7 d timepoints warrant further experiments. Additionally, 7 d timepoints may need to be modeled using an ellipsoidal rather than spherical single-shell model, which is complex and outside the scope of this study. Therefore, all additional characterization conducted in this study assessed the baseline, untreated control, and tenogenically differentiating MSCs at 3 d. Taken together with the detection of significant differences at 3 d, this finding also further narrows the time range in which cell separation may be optimal, as earlier (day 0) and later (day 7) timepoints do not have distinct patterns in crossover frequencies.

### 3.2. Variance in Cell Size

Image analysis using ImageJ resulted in the cell dimensions summarized in Table 2. Baseline cells were spherical, and this sphericity was retained in both 3 d–no treatment controls and 3 d–TGFβ2 treatment groups. These findings are in agreement with a previous study, where cell morphology remained spherical until day 4 in culture [60]. Unpaired t-tests showed no significant differences (*p* = 0.95) in the radii of 3 d–no treatment controls and 3 d–TGFβ2 treated groups. However, the radii of 3 d–no treatment controls and 3 d–TGFβ2 groups differed significantly from the baseline cell radii (*p* < 0.01), indicating that time in culture affected cell size.

The 3 d–no treatment controls and TGFβ2-treated MSCs remained similar in size and shape and exhibited different crossover frequencies (Table 1), signifying a change in membrane and cytoplasmic protein expression with tenogenesis. Thus, the unique DEP properties associated with tenogenic differentiation of MSCs at 3 d are not due to a change in size. Smaller cells usually exhibit lower first crossover frequency, as shown in Equation (5), where *f_x_*_1_ is inversely proportional to the cell radius. When Equation (5) is applied to 3 d–no treatment controls and 3 d–TGFβ2-treated MSCs, they should exhibit lower first crossover frequency due to their large cell radii if their dielectric properties had remained similar. On the contrary, both 3 d–no treatment controls and 3 d–TGFβ2 treatments exhibited different crossover frequency values corresponding to differences in their dielectric properties, despite no significant differences between radii of 3 d–no treatment controls and 3 d–TGFβ2 treatment. This finding illustrates the effectiveness of DEP for detecting and characterizing MSCs based on tenogenic differentiation.

### 3.3. Quantification of Dielectric Properties

#### 3.3.1. Initial Estimates for Modeling

Initial estimates and their bounds impact the final estimated values. Hence, accurate initial estimations significantly reduce the overall time of evaluating the absolute dielectric property values. An inaccurate estimate might result in errors, such as negative R^2^ or R^2^ > 1, infinite, or imaginary values. It is also possible to have final calculated values outside the bounds, which might not be true (physiologically relevant) values. All the initial values are tabulated in Table 3. In an effort to estimate the best-fit values with minimal residual error, bounds are fixed to the initial values.

#### 3.3.2. Modeling of Membrane Properties

Membrane dielectric properties were initially estimated using Equations (5) and (6) and were adjusted for best fit to obtain the mean values and respective standard error (SE). A nominal membrane thickness of 7 nm was used to quantify the dielectric properties of the membrane, as this thickness represents a wide range of stem cells [45,63].

The estimated electrical properties of the membrane are tabulated in Table 4. A decreasing trend is observed for both the permittivity and capacitance of all the three groups. Although the radial changes between 3 d–no treatment controls and 3 d–TGFβ2-treated MSCs are not significantly different from each other in terms of size and sphericity, the 3 d–TGFβ2-treated MSCs expressed higher $f_{x1}$ values (Table 1), resulting in lower permittivity and capacitance. This difference in membrane capacitance and permittivity might be due to the onset of tenogenesis or an increased rate of change in the membrane’s protein expression following the treatment of MSCs with TGFβ2. We previously found that TGFβ2-induced tenogenesis in MSCs was associated with significant changes in the protein levels of the transmembrane cell–cell junction proteins, N-cadherin, Cadherin-11, and Connexin-43 [50]. These protein level changes may impact the membrane properties and account for the unique membrane capacitance, permittivity and $f_{x1}$ with tenogenesis, but this possibility will be explored in future studies.

#### 3.3.3. Modeling of Cytoplasmic Parameters

Following the procedure described in Section 2.4.1, cytoplasmic properties were quantified using the experimentally obtained $f_{x2}$ as tabulated in Table 5. The second crossover frequency is susceptible to ion leakage and less sensitive to the permittivity of the cell’s interior [45]. $f_{x2}$ is also sensitive to temperature changes in the DEP medium solution [49] and lag time between collecting the cultured MSCs and suspending them in the DEP medium. Experimental values of $f_{x2}$ were within ±5 MHz, and the technical (*n* = 9) and biological replicates (*n* = 3) for each group did not change when the time lag between suspending MSCs in DEP media and the DEP experiment was under 30 min, and the exposure times to electric fields remained under a minute (Table 1 and Table 5).

**Figure 6:**
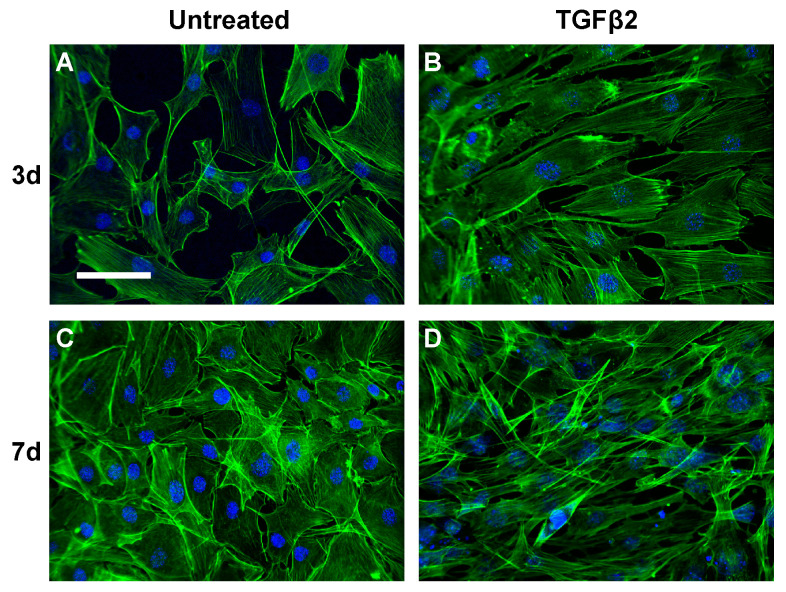
Representative images (20X) of untreated MSCs and MSCs treated with TGFβ2. The actin cytoskeleton (green) and cell nuclei (blue) are shown at 3 and 7 days. (**A**) Untreated cells at day 3 and (**C**) day 7 display typical MSC morphology. (**B**) Cells treated with 50 ng/mL TGFβ2 show increased elongation and proliferation at day 3, and (**D**) these changes become more pronounced at day 7. Scale bar = 100 μm.

Statistical analysis of $f_{x2}$ for the baseline MSCs and the 3 d–no treatment control MSCs showed no significant differences (*p* > 0.05), which is a trend that is also observed for cytoplasmic conductivity. 3 d–TGFβ2-treated MSCs exhibited higher $f_{x2}$ than 3 d–no treatment control and baseline MSC groups. This is because neither size nor shape impacts $f_{x2}$, as illustrated by Equation (7), where there is no dependency on the geometry of the cell [43]. Lower conductivity and increased permittivity of 3 d–TGFβ2-treated MSCs might indicate the onset of tenogenesis and associated cytoplasmic changes. These cells become more polarizable, signifying cellular heterogeneity in regard to the cytoplasm. Additionally, extensive morphological changes in the cell cytoskeleton are seen in tenogenically differentiating MSCs (Figure 6), possibly accounting for the significant differences detected in $f_{x2}$ with 3 d–TGFβ2 treatments. Future studies will explore the specific cytoplasmic contributors to the unique DEP properties of tenogenically differentiating MSCs.

### 3.4. Effect on Clausius–Mossotti Factor as a Function of Frequency

**Figure 7:**
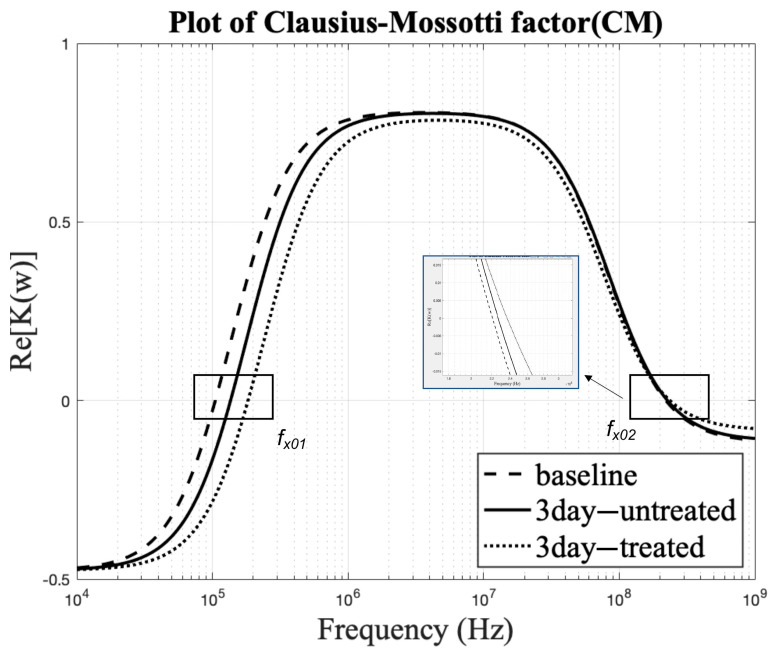
DEP characteristic plot of Re[K(ω)] vs. frequency identifying the first and second crossover frequency, i.e., the coordinates at which the zero line intersects the curves, provides the crossover frequency value.

A plot of Re[K(ω)] over the frequency range, 10^4^–10^9^ Hz (10 kHz–1000 MHz), was generated to understand better the DEP characteristic behavior over a wide frequency range. All the positive y-axis values represent the pDEP behavior of the MSCs, and all the negative y-axis values represent the nDEP behavior of the MSCs. Crossover frequency values can also be estimated using the zero DEP line, i.e., the y-axis at 0, thereby calculating the dielectric properties. Dielectric properties can also be evaluated using Re[K(ω)] values [64]. Figure 7 aids in determining the sorting zone using AC fields to efficiently separate the cells based on their distinct size, shape, and dielectric properties. Frequencies in the positive region of the curve can be used to sort the cells based on the regions where they exhibit distinct behavior, which is indicated through distinctly non-intersecting curves. The inset image is a zoomed-in version at frequencies close to *f_x_*_2_. The curves appear to be merging, which is not the actual case, and hence an inset is used to describe the differences that signify the distinct characteristics of the cytoplasm. However, the high frequencies required to detect *f_x_*_2_ may damage the membrane structure and compromise cell viability. Therefore, studies of *f_x_*_2_ may be more appropriate for end-point MSC characterizations rather than sorting. Future label-free DEP-based sorting devices can be developed based on the unique $f_{x1}$ of tenogenically differentiating MSCs that we determined here.

### 3.5. Comparison of the Theoretical Model to Experimental Frequencies

**Figure 8:**
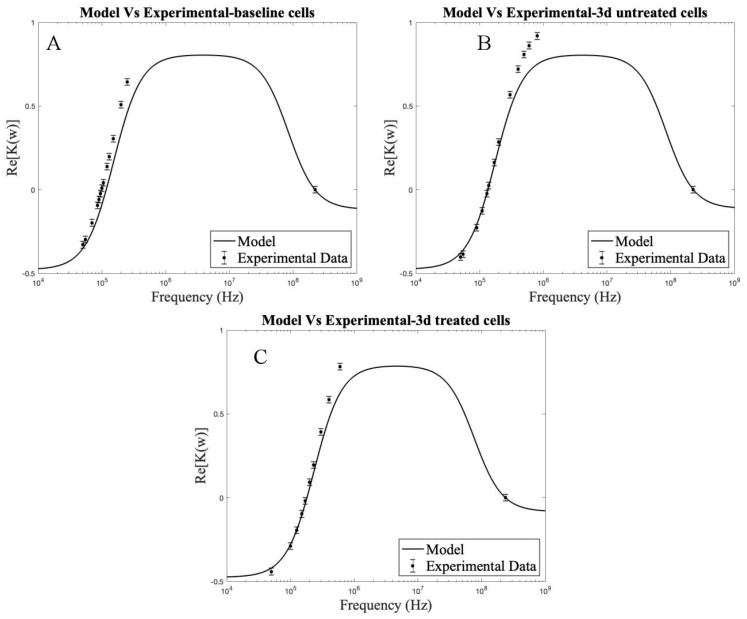
Plots comparing theoretical single shell spherical model to the experimentally determined Re[K(ω)], (**A**) Baseline cells, (**B**) 3 d untreated cells, (**C**) 3 d treated cells. Horizontal error bar of ±5 kHz for $f_{x1}$ is included, and the respective error in Re [K(ω)] (y-axis) is represented.

To compare the accuracy of the theoretical model, the following plots (Figure 8) are included. The fit of the theoretical curve to the experimental Re[K(ω)] is determined from the different frequencies at which experiments were run for all the cell groups, i.e., baseline, 3 d–no treatment control, and 3 d–TGFβ2-treated MSC groups. The data points start to skew as the frequency is increased due to experiments being conducted at a single DEP suspension medium conductivity (0.06 S/m). For more accurate models, experiments at different medium conductivities should be done with subsequent curve-fitting to accurately determine the parameters to be used in the theoretical model.

### 3.6. Cell Viability and Throughput

Although prolonged exposure to electric fields affects cell viability, i.e., within the frequency range of 0.01–1 MHz for a prolonged duration (5–30 min) [18], shorter exposure times of 30 s to 2 min do not substantially affect cell viability or metabolism [65]. Additionally, while FACS and MACS offer limited throughput of ≈5000 cells/s and 280,000 cells/s respectively [66], DEP-assisted sorting devices can expediently sort cells at clinically relevant scales (≈10^9^ cells) using at least four passages at 150,000 cells/h of sorting throughput per passage [66]. Recent work reports throughputs of up to 10,000 cells/s [67,68]. Taken together, prior studies utilizing DEP have shown that it is an effective and efficient label-free technique for generating homogeneous populations of stem cells for tissue engineering applications, with considerable advantages over traditional sorting methods, such as FACS and MACS.

### 3.7. Limitations

This study has some limitations. While tenogenic initiation was evaluated based on changes in cell morphology that match those observed in prior studies [50,69], production of tenogenic marker proteins, scleraxis, and tenomodulin, were not assessed due to the short time points. Our prior work demonstrated that scleraxis and tenomodulin are not detectable at the protein level until at least after 7 days of TGFβ2 treatment [50,69]. It is possible that the morphology of the TGFβ2-treated cells resulted in the different crossover frequencies, as cell shape may impact dielectric properties. To explore how cell morphology, independent of tenogenic induction, impacts crossover frequency, future studies will use other chemical factors to change cell shape without initiating tenogenesis. Notably, the observed differences in crossover frequency correlated with the addition of TGFβ2 at early time points (e.g., 3 d), while cell morphology changes were relatively minimal, suggesting that the initiation of tenogenesis impacts the dielectric properties of the MSCs. Additionally, the changes in crossover frequency may be the result of TGFβ2 altering the membrane or cytoplasmic potential of MSCs via a mechanism unrelated to tenogenesis. Future studies will be needed to explore the possible mechanisms driving the altered dielectric properties of MSCs when treated with TGFβ2.

Finally, this study did not directly compare an affinity-based sorting method, such as FACS or MACS, with DEP. As our goal was to establish the potential of using DEP as a future sorting technique for tenogenically differentiating MSCs, direct comparison was outside the scope of this study. Future work will use the DEP parameters established in this study to quantify the potential advantages of a DEP-based microfluidic sorting device over FACS or MACS for selecting MSCs with the highest tenogenic potential.

### 3.8. Future Directions

Our results using a point-and-planar electrode DEP microwell device confirm the need for further exploration. Future studies based on these findings will develop a DEP-based microfluidic sorting device to isolate and enrich populations of tenogenically differentiating MSCs at 3 d and then explore impacts of DEP purification on tenogenic markers (scleraxis and tenomodulin) in longer-term culture. Further studies in vitro and in vivo are required to characterize cell viability following DEP and initial tenogenesis and later stages, when fully differentiated into tendon cells. Future studies are also needed to better understand the electrophysiology associated with changes in the cytoplasm and membrane throughout tenogenesis. For potential clinical translation, the dielectric properties of human primary MSCs undergoing tenogenesis will be evaluated in future studies.

## 4. Conclusions

DEP has the potential to be developed into a label-free separation technique that can enrich and isolate stem cells effectively and enhance their efficacy for treating injuries and diseases. The distinct dielectric properties of murine MSCs identified in this study reveal their biophysical identities, which can be used to separate cells based on minute differences in their intrinsic electrical properties. Specifically, this study identified a difference in the membrane dielectric properties between day 3–no treatment (undifferentiated control) MSCs and day 3 tenogenically differentiating MSCs treated with TGFβ2. This study represents a significant contribution to DEP ultra-high frequency cytoplasmic characterization of MSCs. Since the higher frequency ranges are not easily accessible, the DEP second crossover regime remains largely unexplored. However, using DEP higher crossover frequency characterizations, cytoplasmic changes can be identified and further studied to enhance understanding of the subsequent changes within the cell nucleus and its content.

Taken together, this study characterized MSCs and their tenogenically differentiating progeny to obtain the dielectric properties. The results and techniques described can be expanded to develop a stem cell sorter for the efficient sorting of differentiating/differentiated MSCs from non-viable cells and undifferentiated cells in a label-free and inexpensive way. The experiments demonstrated a significant difference in the dielectric properties between treatment groups, which further needs to be compared with another characterization technique other than dielectrophoresis, such as marker-based assay, cytometry, for further scale-up and viability studies. Ultimately, stem cell sorting by DEP has the potential for clinical applications because it can improve the homogeneity of stem cell populations and reduce the potential for aberrant tissue formation (e.g., ectopic bone formation). Further studies will continue to develop DEP-based-stem cell sorting and scale it up to the cell numbers needed for regenerative therapies, transplantation, or engineered organs and tissues.

## Figures and Tables

**Table 1:** Table reporting the stem cell first and second crossover experiment values for baseline and days 0, 3, and 7. Day 7 cells experienced extreme heterogeneity consisting of small, large, and elongated cell populations. The medium conductivity for all the experiments was maintained at 0.06 S/m.

Timepoint	Treatment	No Treatment
First Crossover Frequency (*f_x_*_1_)		
Baseline	95–100 kHz	
Day 0 (8 V_pp_)	180–220 kHz	200–210 kHz
Day 3 (8 V_pp_)	165–190 kHz	128–140 kHz
Day 7 (8 V_pp_)	110–130 kHz (small); 50–70 kHz (elongated); 150–175 kHz (big)	135–150 kHz (small); 250–300 kHz (big)
Second Crossover Frequency (*f_x_*_2_)		
Baseline	225–230 MHz	
Day 0 (13 dBm)	215–230 MHz	200–205 MHz
Day 3 (13 dBm)	235–240 MHz	220–230 MHz
Day 7 (13 dBm)	240 MHz	215 MHz

**Table 2:** Various studies reporting sizes of MSCs derived from different sources.

Radius (In μm) of Mesenchymal Stem Cells					
		Baseline (Control)	3 d–No Treatment	3 d–Treatment	Reference
Current study (murine)		8.91 ± 0.091	10.10 ± 0.19	10.11 ± 0.21	
Adams et al. (human)		13.20			[18,45]*
Velugotla et al. (human)	H1-MSCs	7.53 ± 1.55			[61]
	H9-MSCs	6.25 ± 1.14			
Liu et al. (murine)		8.67 ± 0.95			[60]

* Adams et al. 2014 do not cite size in their article. The radius was calculated and reported by [32] based on Adams et al. 2014.

**Table 3:** Initial estimates of dielectric parameters used prior to non-linear regression for obtaining the best-fit parameters by minimizing the residual error; values for the membrane are obtained from [18], and cytoplasmic conductivity range is modified to 0.30–3.0 based on prior studies [48,62].

Cell Component	Permittivity	Conductivity (S/m)
Membrane	6.5–11	10^−3^–10^−8^
Cytoplasm	50–100	0.3–1.5

**Table 4:** Estimated membrane electrical properties using DEP spherical single-shell model, mean ± standard error (SE) are reported for all the cell groups.

Property	Control	3 d–No Treatment	3 d–Treatment
Whole-cell capacitance (pF)	3.83 ± 0.012	3.19 ± 0.08	2.46 ± 0.1
Relative permittivity	3.03 ± 0.01	1.97 ± 0.05	1.51 ± 0.06

**Table 5:** Cytoplasmic properties obtained through the fitting procedure described in Section 2.4.1 using Equation (7), where the 3d–treatment group shows a decrease in cytoplasmic conductivity.

Cell Group	Conductivity (S/m)	$\mathrm{Permittivity}\;(\varepsilon_{cell}/\varepsilon_{0})$
**Baseline (control group)**	0.88 ± 0.01	55 ± 2
**3 d–no treatment**	0.88 ± 0.02	55 ± 1
**3 d–treatment**	0.82 ± 0.02	62 ± 1

## Data Availability

Not applicable.
